# Apoptosis drives cancer cells proliferate and metastasize

**DOI:** 10.1111/j.1582-4934.2012.01663.x

**Published:** 2013-01-11

**Authors:** Rui-An Wang, Qin-Long Li, Zeng-Shan Li, Ping-Ju Zheng, Hui-Zhong Zhang, Xiao-Feng Huang, Su-Min Chi, An-Gang Yang, Rutao Cui

**Affiliations:** a State Key Lab of Tumor Biology, The Fourth Military Medical UniversityXi'an, China; b Department of Pathology of Xijing Hospital, The Fourth Military Medical UniversityXi'an, China; c Department of Pathology and Pathophysiology, The Fourth Military Medical UniversityXi'an, China; d Department of Physiology, The Fourth Military Medical UniversityXi'an, China; e Department of Immunology, The Fourth Military Medical UniversityXi'an, China; f Department of Laboratory Medicine of Tangdu Hospital, The Fourth Military Medical UniversityXi'an, China; g Department of Medical Oncology, Chang'an HospitalXi'an, China; h Department of Dermatology, Boston UniversityBoston, MA, USA

**Keywords:** cancer, apoptosis, cell cycle, metastasis, inflammation, stroma

## Abstract

Cancer has been considered to be the result of accumulated gene mutations, which result in uncontrolled cell proliferations for a long time. Cancers are also regarded to be capable of immune evasion. Furthermore, resistance to apoptosis was recognized as an important trait of cancer in the last score of years. However, there are numerous paradoxical issues in this whole set of theory. For example, there is no known set of genes of which mutations are responsible for human cancers. As for the trait of ‘resistance to apoptosis’, the fact is that cancer has increased frequency of apoptosis. The more malignant the tumour is, the more apoptosis shows. In this study, we propose a new theory that apoptosis plays a key role in the malignant progression and metastasis of cancer. The growth of tumour is the difference between tumour cell proliferation and attrition plus the hyperplastic growth of stroma. Increased and unpreventable death caused by innate or environmental factors such as ischaemia and inflammation drives the tumour cells to proliferate relentlessly, move to new lands to establish colonies. In short, increased cell death is the origin of malignancy.

## Prelude

Life and death is the eternal theme of human society. It is also true in any form of life. Cancer is not an exception.

Cancer has long been regarded as the deregulated cell proliferation and failure of cells to die owing to the accumulation of gene mutations. In other words, cancer is a genetic disease. This is the so-called somatic mutation theory (SMT). The millennium review of Hanahan and Weinberg entitled ‘The Hallmarks of Cancer’ put the ‘evasion of apoptosis’ as one of the six traits of cancer [1]. The review is the most cited article in the past decade, and in fact, it has become the bible of cancer researchers. ‘Resistance to apoptosis’, and ‘evasion of immune destruction’ have become deeply rooted within the subconsciousness of cancer scientists. However, there exist numerous paradoxical issues which put the validity of the SMT theory in question [2]. In this study, we will discuss four issues—gene mutation, cell cycle, apoptosis and stromal hyperplasia in cancers, and propose a novel theory of tumour development and metastasis—apoptosis drives the tumour cells proliferate and metastasize.

## There is no consensus set of genes whose mutations are surely the cause of cancer

The final decade of the last century had witnessed the increasing numbers of gene mutations in various types of cancers. Many oncogenes and tumour suppressor genes were identified and found to play important roles in the biology of tumour cells. We supposed that when the whole human genome became decoded, cancer would be totally elucidated and become history. However, although great efforts have been taken to sequence the whole genome of various types of cancers [3], there was little to tell from all those work. There is no single gene, or set of genes whose mutation or combined mutations have been found to be responsible for human cancers in a wide spectrum. In other words, we can say that all these efforts have proved that cancer is not the result of gene mutations. We still have a long way to go before we fully understand cancer.

## The prolonged cell cycle of cancer cells is contrary to many people's beliefs

What cancer affects on the duration of cell cycle is a very tricky question for cancer researchers. An overwhelming majority of people have the wrong conception that ‘the duration of cell cycle of cancer turns short’. In so many reviews concerning the cell cycle controls of cancer published in recent years, the duration alteration was rarely, if ever, talked about. The truth is, the cell cycle of cancer turns longer in most cases, and some of them have no change [4].

The cell proliferation cycle was found in the middle of last century. It was an extensively studied area, with the pulse labelling of H^3^-thymidine autoradiography as the major approach for nearly a score of years. Many studies had revealed similar results that even the fastest growing tumours demonstrated longer cell cycle. At the time, it had already been concluded that the concept of cancer as a disorder of rapid cell proliferation should be discarded [4]. The FACS assay, which is commonly used today for cell cycle study, cannot measure the cell cycle duration.

**Fig. 1:**
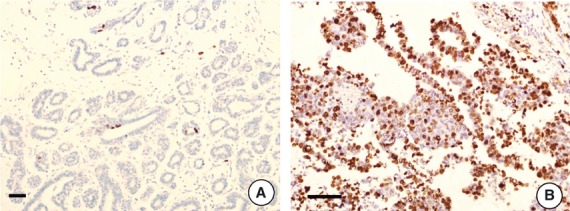
Increased proportion of cells in active proliferation state is responsible for the tumour growth, as shown by the Ki67 immunostaining. (**A**) In normal mammary gland tissue, Ki67 labelling index is quite low, just about 1–2%. (**B**) Invasive breast cancer shows significantly increased Ki67 labelling, about 50%. Bar = 100 μm.

In fact, it is the increased proportion of proliferating cells rather than the speed of cell division that is responsible for the growth of cancer. The ratio of proliferating cells in the total pool of cells was designated as the growth fraction by Mendelsohn [5]. This portion of cells is now easily labelled by the Ki67 immunostaining (Fig. 1). In normal adult tissues, most cells are in quiescent state (G0), which is out of the proliferation cycle. In cancer, this portion of cells is significantly decreased. In the four phases of the cell cycle, S, G2, and M are nearly the same in most types of cells, including both normal and tumour cells. The most variable phase is G1. In cancer cells, this phase is often seen prolonged, called G1 delay. So the add up results is the prolongation of cell cycle. However, the question why the cell cycle of cancerous cells turns longer is still a mystery, or what is the mechanism of G1 delay. Many studies have reported that cancers have increased expression of cyclins such as cyclin D1 and cyclin E, etc. [6]. Cyclin D1 and cyclin E are known to activate the cyclin-dependent kinases (CDK) which further drive the cell cycle from G1 to S phase [5]. Why will cancer cells have G1 delay with the increased cyclin D1 and cyclin E?

The second hallmark of cancer proposed by Hanahan and Weinberg is the ‘insensitivity to growth inhibiting signals’ [1]. Obviously, it contradicts the fact that cancer cells have prolonged cell cycle duration. Although what is responsible for the prolongation is still not known, we suppose it should be some kind of inhibitory mechanism because of such a high proportion of cells entering the cycle. Cyclins are the complementary increases. With the increase in the resistance of the cell cycle, the cyclins have to be increased to drive the cycle through. If the somatic mutations make the cancer cells produce a large amount of cyclins which drive the cells proliferate relentlessly, the cancer cells should have a shorter proliferation cycle. However, we have the reverse fact here.

## Cancers show increased frequency of apoptosis rather than ‘evasion of apoptosis’

Apoptosis has long been regarded as a barrier to carcinogenesis. So for the tumours to arise, it is essential to acquire the ability of ‘resisting apoptosis’. Although we are not sure who exactly initiated this notion, probably it could be accredited to Dr. Korsmeyer for his prominent work in finding and elucidating the role of Bcl-2 [7]. Bcl-2 is a protein that can suppress apoptosis by stabilizing the mitochondria membrane [8]. In the follicular B-cell lymphoma, Bcl-2 is overexpressed owing to the chromosomal translocation t(8,14) which puts the Bcl-2 gene under the control of IgH promoter [7]. Later on many oncogenes were found to be able to suppress apoptosis while stimulating cell proliferation.

**Fig. 2:**
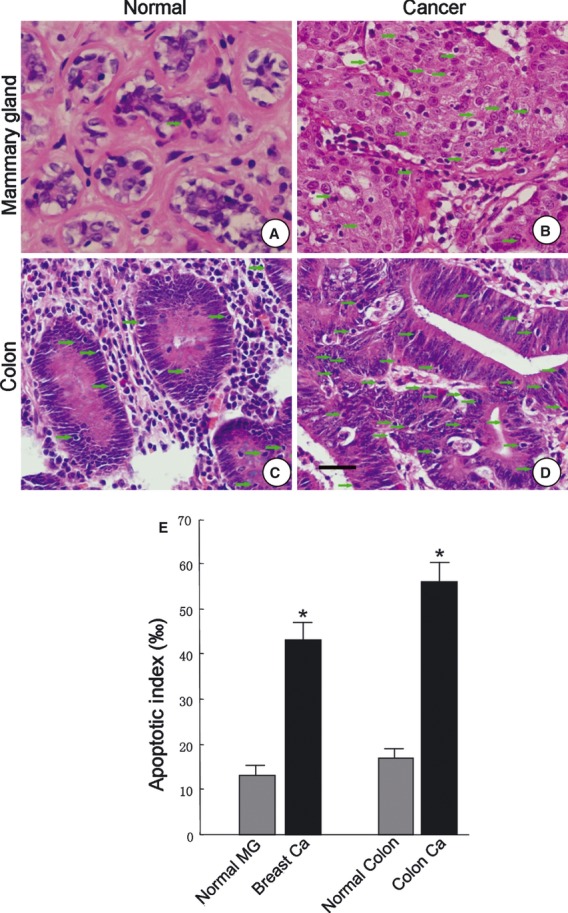
There is much more apoptosis seen in cancerous tissues than in normal tissues, indicated by arrows. It is not difficult for pathologists to pick up apoptosis in H.E stained sections. Bar = 50 μm. (**A**) Normal human mammary gland. (**B**) Breast cancer. (**C**) Normal colon. (**D**) Colon cancer. (**E**) Statistical analysis of the difference between the apoptotic indexes (‰). *N* = 5 in each group. *statistically significant (*P* < 0.05).

‘Evasion of apoptosis’ or ‘resistance to apoptosis’ as a hallmark of cancer has become a doctrine of cancer biology, but there exist quite a few unclear or paradoxical issues. First of all, what's the exact meaning of ‘evasion of apoptosis’? No organism can live forever, neither can cancer cells. ‘Evasion of apoptosis’ means cancer cells live much longer than normal cells from which the cancer originates. It does not mean that cancers have increased apoptosis or any form of death. Ironically the fact is, cancers usually have a significantly increased frequency of apoptosis, *i.e*. the apoptotic index of tumour tissues is much higher than that of normal tissues. The higher the apoptotic index is, the more malignant the tumour is [9–15], which is known to many pathologists, as shown in Figure 2. We also found that a shortened life span of cancer cells was noted by Carrel in 1925. Carrel wrote ‘The malignant monocyte differs from the normal one chiefly because it is a diseased cell which is short lived’ [16]. Fischer later on noted a similar thing that cancer cells were short lived [17].

As far as we know, many pathologists have used the frequency of apoptosis together with the mitotic index to make the differential diagnosis of malignancy or benign, and the degree of malignancy. However, as the ‘evasion of apoptosis’ as a hallmark of cancer has been so popular and has been put into the textbook of pathology, rarely any pathologist openly speaks about it. It is imaginable that in such a situation the pathologist will feel depressive, hopeless and bewildered.

Now the question is why cancer would have led to an increase in apoptosis rather than ‘evasion of apoptosis’? With the increase in apoptosis, how does the tumour develop? These questions sound reasonable, but are as naïve as believing that the sun goes around the earth.

The answer to the above question is simply that the increase in apoptosis is the impetus of the relentless cell proliferation, and thus, the root of malignancy. When studying cancer, we must keep in mind that it is also a form of life. There are universal principles in the circle of life. To keep from distinction, the organism must keep a minimal population. Thus, the higher the death rate is, the higher the birth rate is. Reversely, the longer the organism can live, the lower the birth rate is. The litter size of the surviving rats is increased in the deratization campaign, which is a well-known example. When we chop down a tree, several small trees will grow out from the root. Cancers are abnormal, poorly organized tissue structures inside the body. They face a lot of tough problems, including poor logistic supply as the blood vessels are abnormal and limited, attacks by the immune system, increased gene replication errors and genomic instability. All these issues make the cancer tissues have a high rate of cell loss, and leave them with no choice but to let a high proportion of cells get into the proliferation cycle, as shown by the Ki67 staining. The positive labelling of Ki67 in cancer may be several or dozens of times that of normal tissues (Fig. 1).

**Fig. 3:**
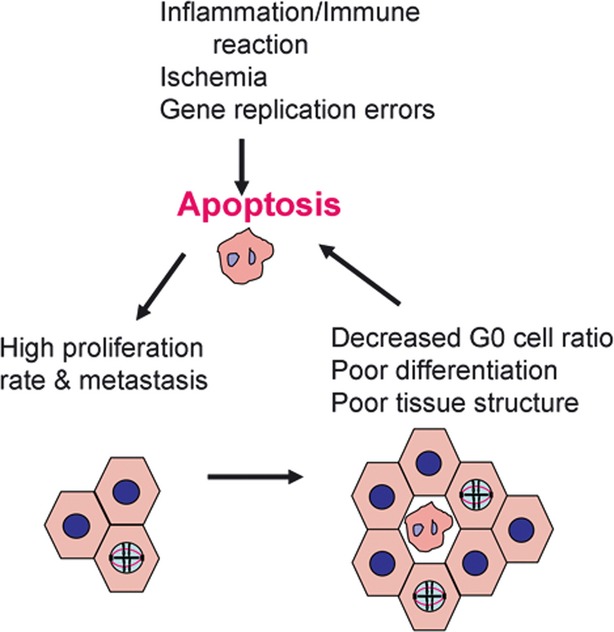
Schematic diagram shows the vicious cycle of high death rate, proliferation and tissue structure.

One principle we must keep in mind is, cells must exit the proliferation cycle to enter the G0 state so as to build organized tissues. So the more the cells die, the less the G0 cell ratio is, and the poorer the tissue differentiation and structure are, which in turn get more cells die (Fig. 3). This is shown as increased apoptosis and sometimes necrosis if an outbreak of apoptosis happens. So basically, cancer is the vicious cycle of death and proliferation of a group of poorly organized cells. In fact, a lot of pathological and animal studies support this notion. The so-called proto-oncogene Bcl-2 is expressed in the normal mammary glandular tissues, as well as in the low- but not in the high-grade breast cancers [10]. Concordantly, high-grade breast cancers have a much higher apoptotic index as well as and proliferation rate [9–12]. And the increased apoptosis or necrosis is linked with shortened survival of the patient [9–12]. The same results that higher Bcl-2 expression was linked with better differentiation and increased patient survival was also observed in colon and lung cancer [13, 14, 18]. The expression level of Bcl-2 in breast cancer and colon cancer are positively related with the survival time of the patient, that is to say, Bcl-2 is a positive factor for cancer patients [10, 13, 19–24].

The relationship between increased apoptosis and tumour development is also shown in animal studies. In a mammary gland transgenic mice study, Murphy *et al*. found that the overexpression of Bcl-2 delayed the DMBA-induced mammary gland carcinogenesis [25]. Reversely, the apoptosis-inducer molecule CD95 promotes cancer growth [26]. These puzzling results of research in the past can be explained with the new theory that apoptosis is the key to cancer progression.

## High frequency of apoptosis drives cancer metastasize

For so many years of studying the molecular mechanisms of tumour development and metastasis, we have never answered the most basic question of why should the cancer metastasize? What for? Where is the motivation from? Although many studies have elaborately delineated the mechanisms of cell migration and invasion, they may in some way have told how the cancer metastasize, but in no way answered the question of why the cancer should ever metastasize. Gene mutation, overexpression of oncogenes, or loss of function of tumour suppressor genes are not answers to this question.

Actually, the answer to this terribly hard question is just that simple. Just have a brief review of American history and one will get it. The pilgrims from England journeyed to the new land by Mayflower to escape religious persecution. So the simple and acceptable answer to the question of why should cancer metastasize is to look for a better and safer life environment. Mankind or any animals migrate for the same reason. When living environment in a place became harsh, why not move? So the poor and dangerous environment drives the cancer cells to look for new land suitable for their inhabitation. The difference between a malignant and a benign tumour is that the latter is better organized, lives a better life, has lower apoptosis and thus, does not have the drive to move.

Although it is reasonable to think the high apoptosis rate is linked with increased cancer cell proliferation and metastasis, what these growth stimulating signals are and how they are sent off to the surviving cells are still an enigma and awaits us to elucidate. They might be some growth factors secreted in either endocrine or paracrine manners, or might also be some electric signals which are very hard to study.

## Stroma is an unnegligible part of tumour

**Fig. 4:**
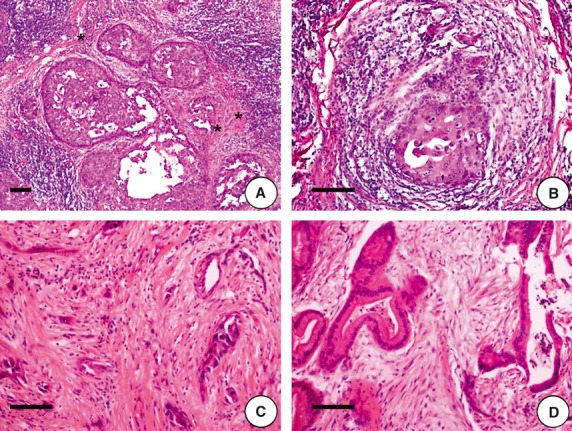
Desmoplastic reaction of stroma and the basement membrane provide defence for the invasive and *in situ* carcinomas. (**A**) Ductal carcinoma *in situ* of breast is surrounded by lymphocytes. However, the basement membrane and collagenous fibres (stars) provide defence against the attack from lymphocytes. (**B**) In contrast with A, the microinvasive cancer cells of breast cancer are being attacked by inflammatory lymphocytes. (**C**) Undifferentiated adenocarcinoma of gallbladder demonstrated prominent desmoplastic reactions, with large amounts of collagenous fibres segregating the cancer cells. (**D**) In a well-differentiated adenocarcinoma of gallbladder, the stroma is close to normal. H.E. staining, bar = 100 μm.

An obvious hallmark of cancer is often the desmoplastic reaction of stroma (Fig. 4). As the normal epithelium has very little interstitial substance and is free of blood vessels and lymphatics, the normal epithelium should have some degree of immune privilege as it is divided from the connective tissue stroma by the basement membrane. It is a pity that our knowledge about the basement membrane is extremely limited. We almost know nothing about its biological function except it provides anchorage for the epithelium.

The cancer stroma should serve at least four functions. First, it provides mechanical support for the cancer tissue structure. Second, it provides logistics to the cancer cells. The angiogenesis of cancer is an issue which is being extensively studied. Third, it provides signals for cancer cells to grow and other activities such as hyaluronan-related cell migration, etc. Finally, it provides defence for the cancer cells against the immune system's attacks. The first two functions of the cancer stroma are so obvious, and the third one is also well known. What we need to discuss more in detail is the defence functions which the stroma serves.

There are several issues between immune/inflammation reaction of the organism and the development, progression and metastasis of the cancer. For most of the time in the last century, the inflammation was considered the defence mechanism of the body, and associated with good prognosis. However, for the last decade, it was gradually realized that chronic inflammation promoted carcinogenesis and, an inflammatory environment nurtured the progression, facilitated the metastasis of cancer [27, 28]. Many studies reported that cytokines secreted by the infiltrated lymphocytes stimulates the growth of tumour cells [29]. The pendulum has swung from one side to another. Can we conciliate the two opposite points of views, and where is the pivot?

It may be naïve to think inflammation will kill the tumour and so the patient will have a good prognosis. However, it would be insane to believe that the organism sends the inflammatory cells to feed the tumour cells to grow and metastasize. Surely, the inflammation is the defence mechanism of the body to eliminate a tumour. If the tumour is small and the immunity is strong enough, then the tumour is eliminated. However, after the tumour has overcome the initial stage, the partial killing from the immune reaction would stimulate the further growth and metastasis of the tumour, as we discussed above. So the promotion of growth and metastasis of tumour by inflammation is an indirect effect. If the killing by immune reaction is reduced as in the situation of CD95 knockdown, the tumour growth would also be compromised. Reversely, overexpression of CD95/Fas promotes the growth of tumour [26]. While a high level of Fas expression was found to be associated with extremely poor clinical outcome in renal cell carcinoma patients [30].

Where there is offence, there must also be defence. The tumour must have a defence system which could minimize the damage brought by the body's immune system. The components of this system include: (1) down-regulation of MHC-I molecules in the tumour cells, by which the tumour cells make themselves difficult for the immune system to identify and attack [31]; (2) secretion of TGF-β and other immune inhibition molecules which counterattack the strikes from the lymphocytes [31]; and (3) establishment of physical barriers which blocks the approach of immune cells to the tumour cells. These physical barriers are widely seen in tumour tissues, such as collagenous materials and basement membrane like materials (Fig. 4). Because these materials are hard to study in *in vitro* system, it is an under researched area.

## Implications of the proposed theory in cancer research and clinical practice

The theory of cancer progression and metastasis we proposed in this study is more rational than SMT theory in understanding the biology of cancer for both cancer researchers and clinical doctors. It explains why the increased apoptosis is related to higher histological grade of tumour malignancy and poorer clinical outcome. It explains why the apoptosis suppressor Bcl-2 is connected with better prognosis in breast, lung and colon cancers. It explains why Fas promotes tumour growth, and why the overexpression of Fas is related to extremely poor prognosis in renal cancer patients.

Furthermore, clinical doctors should be aware of the other side of chemotherapy. In addition to the commonly known adverse effects of the chemotherapy, they should also realize that chemotherapy has the potential to upgrade the malignancy of the tumour and promote its metastasis. For example, anti-angiogenesis therapy reduces the tumour volume by depriving of the nutrition of cancer cells, so this approach of cancer treatment will stimulate the cancer metastasis. However, neurogliomas are good candidates for anti-angiogenic therapies as they do not metastasize.

In conclusion, we have proposed a new theory of tumour progression and metastasis. The key point of the new theory is increased apoptosis of cancer tissues that drives the surviving cancer cells proliferate in an uncontrolled way, and move to new lands to establish more colonies. So far, we almost know nothing about how the cell death stimulated the cell proliferation. In addition, stroma is an important part in both the structure and progression of the tumour, and thus, awaits more intensive study to elucidate its roles in cancer development.

## The end

Life and death is a cycle. If there is no death, then there is no birth. If death is increased, the cycle speeds up.
